# Tetra­kis(1,3,4,6,7,9-hexa­aza-1*H*-phen­alen-6-ium) sodium(I) penta­kis(tetra­fluorido­borate)

**DOI:** 10.1107/S1600536808030286

**Published:** 2008-09-24

**Authors:** Yuqin Jiang, Yanhui Hou, Yongsheng Chen

**Affiliations:** a Key Laboratoy for Functional Polymer Materials and Center for Nanoscale Science and Technology, Institute of Polymer Chemistry, College of Chemistry, Nankai University, Tianjin, Weijin Road No. 94, Tianjin, People’s Republic of China

## Abstract

In the title compound, Na^+^·4C_7_H_5_N_6_^+^·5BF_4_^−^, the Na^+^ ion lies on a fourfold rotation axis and one of the tetra­fluoridoborate ions lies on a site of symmetry 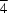. Each Na^+^ ion is surrounded by four symmetry-related tetra­fluoridoborate ions, and is eight-coordinated by F atoms, the Na⋯F separation being 2.3956 (15) or 2.4347 (17) Å. The hexa­azaphenalenium ring system is essentially planar. In the crystal structure, the cations and anions are linked into a three-dimensional network by N—H⋯N and C—H⋯F hydrogen bonds.

## Related literature

For general background, see: Goto *et al.* (1999 ▶); Haddon (1975 ▶); Koutentis *et al.* (2001 ▶). For related structures, see: Morita *et al.* (2002 ▶, 2003 ▶); Tamaki *et al.* (1997 ▶); Zheng *et al.* (2003 ▶, 2005 ▶). For related preparation, see: Suzuki *et al.* (2005 ▶).
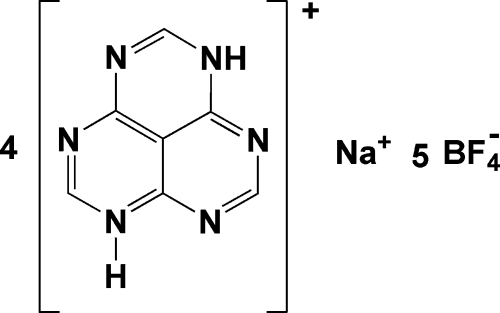


## Experimental

### 

#### Crystal data

Na^+^·4C_7_H_5_N_6_^+^·5BF_4_^−^
*M* *_r_* = 1149.72
Tetragonal, 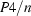

*a* = 15.0665 (6) Å
*c* = 8.9322 (5) Å
*V* = 2027.60 (16) Å^3^
*Z* = 2
Mo *K*α radiation
μ = 0.20 mm^−1^
*T* = 113 (2) K
0.14 × 0.12 × 0.12 mm

#### Data collection

Rigaku Saturn CCD area-detector diffractometer
Absorption correction: multi-scan (*CrystalClear*, Rigaku/MSC, 2005 ▶) *T*_min_ = 0.877, *T*_max_ = 0.977
21698 measured reflections
2423 independent reflections
2247 reflections with *I* > 2σ(*I*)
*R*_int_ = 0.044

#### Refinement

*R*[*F* ^2^ > 2σ(*F* ^2^)] = 0.055
*wR*(*F* ^2^) = 0.152
*S* = 1.19
2423 reflections
177 parameters
H-atom parameters constrained
Δρ_max_ = 0.74 e Å^−3^
Δρ_min_ = −0.79 e Å^−3^

### 

Data collection: *CrystalClear* (Rigaku/MSC, 2005 ▶); cell refinement: *CrystalClear*; data reduction: *CrystalClear*; program(s) used to solve structure: *SHELXS97* (Sheldrick, 2008 ▶); program(s) used to refine structure: *SHELXL97* (Sheldrick, 2008 ▶); molecular graphics: *SHELXTL* (Sheldrick, 2008 ▶); software used to prepare material for publication: *CrystalStructure* (Rigaku/MSC, 2005 ▶).

## Supplementary Material

Crystal structure: contains datablocks global, I. DOI: 10.1107/S1600536808030286/ci2666sup1.cif

Structure factors: contains datablocks I. DOI: 10.1107/S1600536808030286/ci2666Isup2.hkl

Additional supplementary materials:  crystallographic information; 3D view; checkCIF report

## Figures and Tables

**Table 1:** Hydrogen-bond geometry (Å, °)

*D*—H⋯*A*	*D*—H	H⋯*A*	*D*⋯*A*	*D*—H⋯*A*
N3—H3*N*⋯N4^i^	0.88	2.05	2.930 (2)	176
N6—H6*N*⋯N5^ii^	0.88	2.09	2.866 (2)	146
C1—H1⋯F3^iii^	0.95	2.47	3.179 (2)	132
C1—H1⋯F4^ii^	0.95	2.55	2.940 (3)	105
C3—H3⋯F2^i^	0.95	2.22	3.124 (2)	159
C5—H5⋯F3^iv^	0.95	2.40	3.097 (2)	130

Symmetry codes: (i) 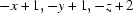; (ii) 
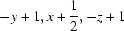
; (iii) 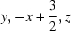; (iv) 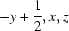.

**Fig. 1.**
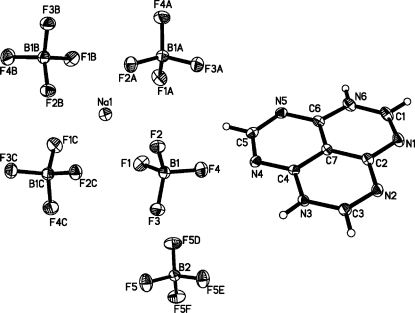
A view of the environment around the Na+ ion in the title compound. Displacement ellipsoids are drawn at the 50% probability level. Atoms labelled with the suffixes A, B, C, D, E and F are generated by the symmetry operations (y, 1/2-x, z), (1/2-x, 1/2-y, z), (1/2-y, x, z), (1-y, x-1/2, -z), (y+1/2, 1-x, -z) and (3/2-x, 1/2-y, z), respectively.

**Fig. 2.**
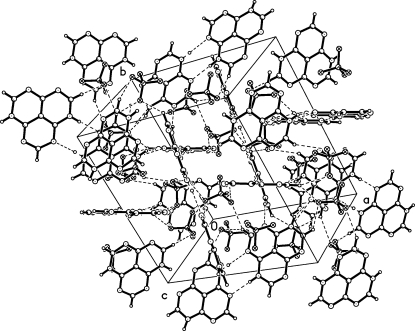
The crystal packing of the title compound.

## supplementary crystallographic information

### Comment

Phenalenyl, as a planar π system with D_3_*_h_* symmetry, has received much attention and still played an important role as a building block for spin-mediated molecular functional materials (Haddon 1975; Koutentis *et al.*, 2001; Goto *et al.*, 1999). Hexaazaphenalene (HAP) is a highly symmetric heterocycle with full nitrogen substitution in all of the α sites of phenalene (Morita *et al.*, 2002; Morita *et al.*, 2003; Suzuki *et al.*, 2005; Tamaki *et al.*, 1997; Zheng *et al.*, 2003; Zheng *et al.*, 2005). Because of the directionality of lone-pair electrons at the nitrogen sites, incorporation of nitrogen atoms into the phenalenyl skeleton was found to give substantial effects on its electronic structure. Suzuki *et al.* have reported the preparation and single cystal structure of the anion of hexaazaphenalene. Herein, we report the preparation and crystal structure of a hexaazaphenalene fluoroboric salt, the title compound.

In the title compound, the Na^+^ ion lies on a four-fold rotation axis and one of the tetrafluoridoborate ions lies on a 4 site symmetry. One of the remaining four BF_4_^-^ ions and one of the four hexaazaphenalenium ions are symmetry independent, and they lie on general positions. Each Na^+^ ion is surrounded by four symmetry related tetrafluoridoborate ions, with the Na···F separation being 2.3956 (15) or 2.4347 (17) Å (Fig.1). The hexaazaphenalenium ring system is essentially planar, with a maximum deviation of 0.042 (2) Å for atom N6. The C—N distances lie in the range 1.310 (3)-1.363 (3) Å.

Two types of hydrogen bonds viz. N—H···N and C—H···F exist in the solid state of the title compound. These hydrogen bonds link the cationic and anionic units into a three-dimensional network (Fig. 2). There are no face to face π-π staking involving the hexaazaphenalenium units, which is a common packing mode in other phenalene compounds.

### Experimental

The sodium salt of hexaazaphenalene was prepared according to the literature method (Suzuki *et al.*, 2005). To a water (5 ml) solution of sodium salt of hexaazaphenalene (97 mg, 0.5 mmol) was added 40% HBF_4_ (0.5 ml, 3.0 mmol). The reaction mixture was evaporated slowly for 7 d to afford colourless crystals of the title compound.

### Refinement

H atoms were initially located in difference maps and then refined using the riding-model approximation, with C-H = 0.95 Å, N-H = 0.88 Å and U_iso_(H) = 1.2U_eq_(C,N).

### Crystal data

**Table:**

Na^+^·4C_7_H_5_N_6_^+^·5BF_4_^−^	*D*_x_ = 1.883 Mg m^−^^3^
*M**_r_* = 1149.72	Mo *K*α radiation, λ = 0.71070 Å
Tetragonal, *P*4/*n*	Cell parameters from 4230 reflections
Hall symbol: -P 4a	θ = 1.9–27.9°
*a* = 15.0665 (6) Å	µ = 0.20 mm^−^^1^
*c* = 8.9322 (5) Å	*T* = 113 K
*V* = 2027.60 (16) Å^3^	Block, colourless
*Z* = 2	0.14 × 0.12 × 0.12 mm
*F*(000) = 1144	

### Data collection

**Table:**

Rigaku Saturn CCD area-detector diffractometer	2423 independent reflections
Radiation source: rotating anode	2247 reflections with *I* > 2σ(*I*)
confocal	*R*_int_ = 0.044
Detector resolution: 7.31 pixels mm^-1^	θ_max_ = 27.9°, θ_min_ = 1.9°
ω and φ scans	*h* = −19→19
Absorption correction: multi-scan (CrystalClear, Rigaku/MSC, 2005)	*k* = −19→19
*T*_min_ = 0.878, *T*_max_ = 0.977	*l* = −11→11
21698 measured reflections	

### Refinement

**Table:**

Refinement on *F*^2^	Primary atom site location: structure-invariant direct methods
Least-squares matrix: full	Secondary atom site location: difference Fourier map
*R*[*F*^2^ > 2σ(*F*^2^)] = 0.055	Hydrogen site location: inferred from neighbouring sites
*wR*(*F*^2^) = 0.152	H-atom parameters constrained
*S* = 1.19	*w* = 1/[σ^2^(*F*_o_^2^) + (0.0759*P*)^2^ + 1.4095*P*] where *P* = (*F*_o_^2^ + 2*F*_c_^2^)/3
2423 reflections	(Δ/σ)_max_ = 0.001
177 parameters	Δρ_max_ = 0.74 e Å^−^^3^
0 restraints	Δρ_min_ = −0.79 e Å^−^^3^

### Special details

**Table:**

Geometry. All e.s.d.'s (except the e.s.d. in the dihedral angle between two l.s. planes) are estimated using the full covariance matrix. The cell e.s.d.'s are taken into account individually in the estimation of e.s.d.'s in distances, angles and torsion angles; correlations between e.s.d.'s in cell parameters are only used when they are defined by crystal symmetry. An approximate (isotropic) treatment of cell e.s.d.'s is used for estimating e.s.d.'s involving l.s. planes.
Refinement. Refinement of *F*^2^ against ALL reflections. The weighted *R*-factor *wR* and goodness of fit *S* are based on *F*^2^, conventional *R*-factors *R* are based on *F*, with *F* set to zero for negative *F*^2^. The threshold expression of *F*^2^ > σ(*F*^2^) is used only for calculating *R*-factors(gt) *etc*. and is not relevant to the choice of reflections for refinement. *R*-factors based on *F*^2^ are statistically about twice as large as those based on *F*, and *R*- factors based on ALL data will be even larger.

### Fractional atomic coordinates and isotropic or equivalent isotropic displacement parameters (Å^2^)

**Table:**

	*x*	*y*	*z*	*U*_iso_*/*U*_eq_	
Na1	0.2500	0.2500	0.4845 (2)	0.0275 (4)	
B1	0.42320 (16)	0.33819 (16)	0.5211 (3)	0.0204 (5)	
F1	0.39625 (9)	0.28695 (10)	0.39963 (15)	0.0293 (4)	
F2	0.35132 (8)	0.34700 (9)	0.61981 (15)	0.0241 (3)	
F3	0.49388 (9)	0.29764 (9)	0.59536 (16)	0.0274 (3)	
F4	0.44983 (10)	0.42159 (9)	0.47314 (19)	0.0346 (4)	
F5	0.73061 (11)	0.17735 (10)	0.08990 (17)	0.0371 (4)	
B2	0.7500	0.2500	0.0000	0.0203 (9)	
C1	0.43924 (15)	0.86438 (14)	0.7069 (2)	0.0202 (4)	
H1	0.4317	0.9205	0.6602	0.024*	
C2	0.50050 (13)	0.78212 (13)	0.8918 (2)	0.0150 (4)	
C3	0.56501 (13)	0.69463 (13)	1.0679 (2)	0.0164 (4)	
H3	0.6034	0.6886	1.1517	0.020*	
C4	0.47115 (12)	0.62292 (12)	0.8956 (2)	0.0126 (4)	
C5	0.38121 (13)	0.56354 (13)	0.7195 (2)	0.0157 (4)	
H5	0.3522	0.5127	0.6798	0.019*	
C6	0.40548 (13)	0.71345 (12)	0.7050 (2)	0.0135 (4)	
C7	0.45850 (12)	0.70676 (12)	0.8328 (2)	0.0124 (4)	
N1	0.48868 (12)	0.86262 (11)	0.82849 (19)	0.0188 (4)	
N2	0.55508 (11)	0.77454 (11)	1.01274 (19)	0.0166 (4)	
N3	0.52553 (11)	0.61957 (11)	1.01648 (18)	0.0138 (3)	
H3N	0.5353	0.5686	1.0617	0.017*	
N4	0.43264 (11)	0.54996 (11)	0.83918 (18)	0.0146 (4)	
N5	0.36565 (11)	0.64098 (11)	0.64850 (19)	0.0155 (4)	
N6	0.39805 (12)	0.79459 (11)	0.64176 (19)	0.0181 (4)	
H6N	0.3669	0.8019	0.5594	0.022*	

### Atomic displacement parameters (Å^2^)

**Table:**

	*U*^11^	*U*^22^	*U*^33^	*U*^12^	*U*^13^	*U*^23^
Na1	0.0214 (6)	0.0214 (6)	0.0396 (11)	0.000	0.000	0.000
B1	0.0199 (11)	0.0185 (11)	0.0227 (12)	−0.0013 (9)	0.0011 (9)	0.0025 (9)
F1	0.0278 (7)	0.0369 (8)	0.0231 (7)	−0.0037 (6)	0.0034 (5)	−0.0066 (6)
F2	0.0218 (7)	0.0290 (7)	0.0214 (7)	0.0028 (5)	−0.0002 (5)	−0.0023 (5)
F3	0.0219 (7)	0.0271 (7)	0.0332 (8)	0.0028 (5)	−0.0018 (5)	0.0047 (6)
F4	0.0324 (8)	0.0248 (7)	0.0466 (9)	−0.0042 (6)	−0.0024 (7)	0.0124 (6)
F5	0.0538 (10)	0.0295 (8)	0.0279 (8)	−0.0065 (7)	0.0028 (7)	0.0055 (6)
B2	0.0219 (14)	0.0219 (14)	0.017 (2)	0.000	0.000	0.000
C1	0.0289 (11)	0.0151 (9)	0.0167 (9)	0.0048 (8)	0.0014 (8)	0.0023 (8)
C2	0.0178 (9)	0.0139 (9)	0.0134 (9)	0.0013 (7)	0.0007 (7)	0.0003 (7)
C3	0.0188 (9)	0.0164 (9)	0.0139 (9)	0.0011 (7)	−0.0035 (7)	−0.0004 (7)
C4	0.0115 (8)	0.0136 (9)	0.0126 (8)	0.0013 (7)	0.0004 (7)	0.0005 (7)
C5	0.0131 (8)	0.0185 (9)	0.0155 (9)	−0.0013 (7)	−0.0005 (7)	−0.0013 (7)
C6	0.0159 (9)	0.0147 (9)	0.0099 (8)	0.0026 (7)	0.0003 (7)	−0.0005 (7)
C7	0.0141 (8)	0.0123 (9)	0.0110 (8)	0.0005 (7)	0.0009 (7)	0.0006 (7)
N1	0.0253 (9)	0.0131 (8)	0.0181 (8)	0.0011 (6)	−0.0028 (7)	0.0016 (6)
N2	0.0205 (9)	0.0139 (8)	0.0155 (8)	−0.0003 (6)	−0.0044 (6)	−0.0005 (6)
N3	0.0165 (8)	0.0120 (8)	0.0130 (8)	0.0010 (6)	−0.0029 (6)	0.0023 (6)
N4	0.0144 (8)	0.0153 (8)	0.0141 (8)	−0.0017 (6)	−0.0012 (6)	−0.0001 (6)
N5	0.0143 (8)	0.0177 (8)	0.0146 (8)	0.0006 (6)	−0.0016 (6)	−0.0017 (6)
N6	0.0249 (9)	0.0171 (8)	0.0124 (8)	0.0045 (7)	−0.0018 (7)	0.0033 (6)

### Geometric parameters (Å, °)

**Table:**

Na1—F1^i^	2.3956 (15)	C1—H1	0.95
Na1—F1^ii^	2.3956 (15)	C2—N1	1.350 (3)
Na1—F1^iii^	2.3956 (15)	C2—N2	1.363 (3)
Na1—F1	2.3957 (15)	C2—C7	1.403 (3)
Na1—F2	2.4347 (17)	C3—N2	1.310 (3)
Na1—F2^i^	2.4347 (17)	C3—N3	1.358 (3)
Na1—F2^ii^	2.4347 (17)	C3—H3	0.95
Na1—F2^iii^	2.4347 (17)	C4—N4	1.341 (2)
B1—F4	1.387 (3)	C4—N3	1.356 (2)
B1—F1	1.392 (3)	C4—C7	1.395 (3)
B1—F3	1.395 (3)	C5—N4	1.336 (3)
B1—F2	1.403 (3)	C5—N5	1.349 (3)
F5—B2	1.3887 (14)	C5—H5	0.95
B2—F5^iv^	1.3887 (14)	C6—N5	1.344 (3)
B2—F5^v^	1.3887 (14)	C6—N6	1.351 (2)
B2—F5^vi^	1.3887 (14)	C6—C7	1.397 (3)
C1—N1	1.317 (3)	N3—H3N	0.88
C1—N6	1.353 (3)	N6—H6N	0.88
			
F1^i^—Na1—F1^ii^	84.26 (3)	F1—Na1—B1^iii^	104.13 (6)
F1^i^—Na1—F1^iii^	143.12 (11)	F2—Na1—B1^iii^	72.30 (6)
F1^ii^—Na1—F1^iii^	84.26 (3)	F2^i^—Na1—B1^iii^	140.43 (9)
F1^i^—Na1—F1	84.26 (3)	F2^ii^—Na1—B1^iii^	101.17 (7)
F1^ii^—Na1—F1	143.12 (11)	F2^iii^—Na1—B1^iii^	28.22 (6)
F1^iii^—Na1—F1	84.26 (3)	B1—Na1—B1^iii^	89.293 (14)
F1^i^—Na1—F2	124.30 (5)	B1^i^—Na1—B1^iii^	167.24 (13)
F1^ii^—Na1—F2	150.83 (6)	B1^ii^—Na1—B1^iii^	89.292 (14)
F1^iii^—Na1—F2	75.56 (5)	F4—B1—F1	110.24 (19)
F1—Na1—F2	56.00 (4)	F4—B1—F3	108.83 (18)
F1^i^—Na1—F2^i^	56.00 (4)	F1—B1—F3	110.49 (19)
F1^ii^—Na1—F2^i^	124.30 (5)	F4—B1—F2	109.39 (19)
F1^iii^—Na1—F2^i^	150.83 (6)	F1—B1—F2	108.49 (18)
F1—Na1—F2^i^	75.56 (5)	F3—B1—F2	109.39 (18)
F2—Na1—F2^i^	75.73 (5)	F4—B1—Na1	129.09 (15)
F1^i^—Na1—F2^ii^	75.56 (5)	F1—B1—Na1	53.49 (10)
F1^ii^—Na1—F2^ii^	56.00 (4)	F3—B1—Na1	122.09 (14)
F1^iii^—Na1—F2^ii^	124.30 (5)	F2—B1—Na1	55.16 (10)
F1—Na1—F2^ii^	150.84 (6)	B1—F1—Na1	98.66 (13)
F2—Na1—F2^ii^	120.46 (11)	B1—F2—Na1	96.62 (12)
F2^i^—Na1—F2^ii^	75.73 (5)	F5—B2—F5^iv^	109.54 (6)
F1^i^—Na1—F2^iii^	150.84 (6)	F5—B2—F5^v^	109.54 (6)
F1^ii^—Na1—F2^iii^	75.56 (5)	F5^iv^—B2—F5^v^	109.34 (13)
F1^iii^—Na1—F2^iii^	56.00 (4)	F5—B2—F5^vi^	109.34 (13)
F1—Na1—F2^iii^	124.29 (5)	F5^iv^—B2—F5^vi^	109.54 (6)
F2—Na1—F2^iii^	75.73 (5)	F5^v^—B2—F5^vi^	109.54 (6)
F2^i^—Na1—F2^iii^	120.46 (11)	N1—C1—N6	126.77 (19)
F2^ii^—Na1—F2^iii^	75.73 (5)	N1—C1—H1	116.6
F1^i^—Na1—B1	104.13 (6)	N6—C1—H1	116.6
F1^ii^—Na1—B1	162.64 (8)	N1—C2—N2	119.14 (18)
F1^iii^—Na1—B1	79.99 (6)	N1—C2—C7	120.70 (18)
F1—Na1—B1	27.85 (6)	N2—C2—C7	120.15 (18)
F2—Na1—B1	28.22 (6)	N2—C3—N3	126.03 (18)
F2^i^—Na1—B1	72.30 (6)	N2—C3—H3	117.0
F2^ii^—Na1—B1	140.43 (9)	N3—C3—H3	117.0
F2^iii^—Na1—B1	101.16 (7)	N4—C4—N3	122.01 (17)
F1^i^—Na1—B1^i^	27.85 (6)	N4—C4—C7	122.11 (17)
F1^ii^—Na1—B1^i^	104.13 (6)	N3—C4—C7	115.87 (17)
F1^iii^—Na1—B1^i^	162.64 (8)	N4—C5—N5	127.57 (18)
F1—Na1—B1^i^	79.99 (6)	N4—C5—H5	116.2
F2—Na1—B1^i^	101.16 (7)	N5—C5—H5	116.2
F2^i^—Na1—B1^i^	28.22 (6)	N5—C6—N6	122.77 (18)
F2^ii^—Na1—B1^i^	72.30 (6)	N5—C6—C7	120.22 (17)
F2^iii^—Na1—B1^i^	140.43 (9)	N6—C6—C7	117.00 (17)
B1—Na1—B1^i^	89.292 (14)	C4—C7—C6	118.18 (17)
F1^i^—Na1—B1^ii^	79.99 (6)	C4—C7—C2	121.34 (18)
F1^ii^—Na1—B1^ii^	27.85 (6)	C6—C7—C2	120.43 (17)
F1^iii^—Na1—B1^ii^	104.13 (6)	C1—N1—C2	115.98 (18)
F1—Na1—B1^ii^	162.64 (8)	C3—N2—C2	116.39 (17)
F2—Na1—B1^ii^	140.43 (9)	C4—N3—C3	120.21 (16)
F2^i^—Na1—B1^ii^	101.17 (7)	C4—N3—H3N	119.9
F2^ii^—Na1—B1^ii^	28.22 (6)	C3—N3—H3N	119.9
F2^iii^—Na1—B1^ii^	72.30 (6)	C5—N4—C4	115.22 (17)
B1—Na1—B1^ii^	167.24 (13)	C6—N5—C5	116.66 (17)
B1^i^—Na1—B1^ii^	89.293 (14)	C6—N6—C1	119.05 (18)
F1^i^—Na1—B1^iii^	162.64 (8)	C6—N6—H6N	120.5
F1^ii^—Na1—B1^iii^	79.99 (6)	C1—N6—H6N	120.5
F1^iii^—Na1—B1^iii^	27.85 (6)		
			
F1^i^—Na1—B1—F4	133.1 (2)	F2^i^—Na1—F1—B1	−79.36 (13)
F1^ii^—Na1—B1—F4	15.7 (4)	F2^ii^—Na1—F1—B1	−89.7 (2)
F1^iii^—Na1—B1—F4	−9.4 (2)	F2^iii^—Na1—F1—B1	37.90 (16)
F1—Na1—B1—F4	87.3 (2)	B1^i^—Na1—F1—B1	−107.90 (14)
F2—Na1—B1—F4	−87.5 (2)	B1^ii^—Na1—F1—B1	−160.5 (3)
F2^i^—Na1—B1—F4	179.8 (2)	B1^iii^—Na1—F1—B1	59.75 (12)
F2^ii^—Na1—B1—F4	−142.58 (17)	F4—B1—F2—Na1	124.71 (16)
F2^iii^—Na1—B1—F4	−61.5 (2)	F1—B1—F2—Na1	4.42 (17)
B1^i^—Na1—B1—F4	156.90 (17)	F3—B1—F2—Na1	−116.18 (16)
B1^ii^—Na1—B1—F4	−119.4 (2)	F1^i^—Na1—F2—B1	49.76 (16)
B1^iii^—Na1—B1—F4	−35.8 (2)	F1^ii^—Na1—F2—B1	−143.4 (2)
F1^i^—Na1—B1—F1	45.78 (13)	F1^iii^—Na1—F2—B1	−95.75 (12)
F1^ii^—Na1—B1—F1	−71.6 (4)	F1—Na1—F2—B1	−2.94 (12)
F1^iii^—Na1—B1—F1	−96.70 (14)	F2^i^—Na1—F2—B1	79.09 (14)
F2—Na1—B1—F1	−174.8 (2)	F2^ii^—Na1—F2—B1	142.68 (12)
F2^i^—Na1—B1—F1	92.50 (13)	F2^iii^—Na1—F2—B1	−153.72 (12)
F2^ii^—Na1—B1—F1	130.10 (12)	B1^i^—Na1—F2—B1	66.77 (10)
F2^iii^—Na1—B1—F1	−148.85 (13)	B1^ii^—Na1—F2—B1	169.42 (10)
B1^i^—Na1—B1—F1	69.58 (15)	B1^iii^—Na1—F2—B1	−124.56 (13)
B1^ii^—Na1—B1—F1	153.24 (12)	N4—C4—C7—C6	−1.5 (3)
B1^iii^—Na1—B1—F1	−123.10 (13)	N3—C4—C7—C6	177.86 (16)
F1^i^—Na1—B1—F3	−47.1 (2)	N4—C4—C7—C2	−178.91 (18)
F1^ii^—Na1—B1—F3	−164.5 (2)	N3—C4—C7—C2	0.4 (3)
F1^iii^—Na1—B1—F3	170.43 (18)	N5—C6—C7—C4	2.3 (3)
F1—Na1—B1—F3	−92.9 (2)	N6—C6—C7—C4	−176.63 (17)
F2—Na1—B1—F3	92.3 (2)	N5—C6—C7—C2	179.74 (18)
F2^i^—Na1—B1—F3	−0.36 (17)	N6—C6—C7—C2	0.8 (3)
F2^ii^—Na1—B1—F3	37.2 (2)	N1—C2—C7—C4	179.01 (18)
F2^iii^—Na1—B1—F3	118.28 (17)	N2—C2—C7—C4	0.1 (3)
B1^i^—Na1—B1—F3	−23.3 (2)	N1—C2—C7—C6	1.7 (3)
B1^ii^—Na1—B1—F3	60.38 (17)	N2—C2—C7—C6	−177.25 (18)
B1^iii^—Na1—B1—F3	144.04 (13)	N6—C1—N1—C2	1.5 (3)
F1^i^—Na1—B1—F2	−139.43 (13)	N2—C2—N1—C1	176.21 (19)
F1^ii^—Na1—B1—F2	103.2 (3)	C7—C2—N1—C1	−2.7 (3)
F1^iii^—Na1—B1—F2	78.08 (12)	N3—C3—N2—C2	−0.6 (3)
F1—Na1—B1—F2	174.8 (2)	N1—C2—N2—C3	−179.00 (18)
F2^i^—Na1—B1—F2	−92.71 (14)	C7—C2—N2—C3	−0.1 (3)
F2^ii^—Na1—B1—F2	−55.11 (18)	N4—C4—N3—C3	178.34 (18)
F2^iii^—Na1—B1—F2	25.93 (12)	C7—C4—N3—C3	−1.0 (3)
B1^i^—Na1—B1—F2	−115.63 (11)	N2—C3—N3—C4	1.1 (3)
B1^ii^—Na1—B1—F2	−31.97 (11)	N5—C5—N4—C4	1.6 (3)
B1^iii^—Na1—B1—F2	51.69 (14)	N3—C4—N4—C5	−179.66 (17)
F4—B1—F1—Na1	−124.27 (16)	C7—C4—N4—C5	−0.3 (3)
F3—B1—F1—Na1	115.40 (16)	N6—C6—N5—C5	177.64 (18)
F2—B1—F1—Na1	−4.51 (18)	C7—C6—N5—C5	−1.2 (3)
F1^i^—Na1—F1—B1	−135.69 (13)	N4—C5—N5—C6	−0.8 (3)
F1^ii^—Na1—F1—B1	151.86 (13)	N5—C6—N6—C1	179.05 (18)
F1^iii^—Na1—F1—B1	79.41 (15)	C7—C6—N6—C1	−2.1 (3)
F2—Na1—F1—B1	2.97 (12)	N1—C1—N6—C6	1.0 (3)

Symmetry codes: (i) *y*, −*x*+1/2, *z*; (ii) −*x*+1/2, −*y*+1/2, *z*; (iii) −*y*+1/2, *x*, *z*; (iv) −*y*+1, *x*−1/2, −*z*; (v) *y*+1/2, −*x*+1, −*z*; (vi) −*x*+3/2, −*y*+1/2, *z*.

### Hydrogen-bond geometry (Å, °)

**Table:**

*D*—H···*A*	*D*—H	H···*A*	*D*···*A*	*D*—H···*A*
N3—H3N···N4^vii^	0.88	2.05	2.930 (2)	176
N6—H6N···N5^viii^	0.88	2.09	2.866 (2)	146
C1—H1···F3^ix^	0.95	2.47	3.179 (2)	132
C1—H1···F4^viii^	0.95	2.55	2.940 (3)	105
C3—H3···F2^vii^	0.95	2.22	3.124 (2)	159
C5—H5···F3^iii^	0.95	2.40	3.097 (2)	130

Symmetry codes: (vii) −*x*+1, −*y*+1, −*z*+2; (viii) −*y*+1, *x*+1/2, −*z*+1; (ix) *y*, −*x*+3/2, *z*; (iii) −*y*+1/2, *x*, *z*.
